# Risk factors for antepartum stillbirth and the influence of maternal age in New South Wales Australia: A population based study

**DOI:** 10.1186/1471-2393-13-12

**Published:** 2013-01-16

**Authors:** Adrienne Gordon, Camille Raynes-Greenow, Kevin McGeechan, Jonathan Morris, Heather Jeffery

**Affiliations:** 1 Department of Neonatal Medicine, Royal Prince Alfred Hospital, Sydney, NSW, 2050, Australia; 2 Sydney School of Public Health, University of Sydney, Sydney, NSW, 2006, Australia; 3 Sydney Medical School, University of Sydney, Sydney, NSW, 2006, Australia; 4 Perinatal Research, Kolling Institute of Medical Research, Sydney University, Royal North Shore Hospital, St. Leonards, Sydney, NSW, Australia

**Keywords:** Stillbirth, Maternal age, Risk factors, Population-based, Data linkage

## Abstract

**Background:**

Maternal age is a known risk factor for stillbirth and delayed childbearing is a societal norm in developed country settings. The timing and reasons for age being a risk factor are less clear. This study aimed to document the gestational specific risk of maternal age throughout pregnancy and whether the underlying causes of stillbirth differ for older women.

**Methods:**

Using linkage of state maternity and perinatal death data collections the authors assessed risk factors for antepartum stillbirth in New South Wales Australia for births between 2002 – 2006 (n = 327,690) using a Cox proportional hazards model. Gestational age specific risk was calculated for different maternal age groups. Deaths were classified according to the Perinatal Mortality Classifications of the Perinatal Society of Australia and New Zealand.

**Results:**

Maternal age was a significant independent risk factor for antepartum stillbirth (35 – 39 years HR 1.4 95% CI 1.12 – 1.75; ≥ 40 years HR 2.41 95% CI 1.8 – 3.23). Other significant risk factors were smoking HR 1.82 (95% CI 1.56 –2.12) nulliparity HR 1.23 (95% CI 1.08 – 1.40), pre-existing hypertension HR 2.77 (95% CI 1.94 – 3.97) and pre-existing diabetes HR 2.65 (95% CI 1.63 – 4.32). For women aged 40 or over the risk of antepartum stillbirth beyond 40 weeks was 1 in 455 ongoing pregnancies compared with 1 in 1177 ongoing pregnancies for those under 40. This risk was increased in nulliparous women to 1 in 247 ongoing pregnancies. Unexplained stillbirths were the most common classification for all women, stillbirths classified as perinatal infection were more common in the women aged 40 or above.

**Conclusions:**

Women aged 35 or older in a first pregnancy should be counselled regarding stillbirth risk at the end of pregnancy to assist with informed decision making regarding delivery. For women aged 40 or older in their first pregnancy it would be reasonable to offer induction of labour by 40 weeks gestation.

## Background

Stillbirth is a devastating pregnancy outcome that is estimated to occur in 2.65 million births globally each year [1]. Stillbirth is relatively understudied and the deaths have not been accounted for globally as part of other major maternal and child health strategies [2]. In Australia 1 in every 140 babies are stillborn which represented 2188 families in 2008 [3]. Recent meta analyses of population based studies in high income countries showed that the major risk factors for stillbirth were maternal age, smoking and obesity with a population attributable risk of 30% [4]. The increasing prevalence of these population risk factors has been postulated to contribute to the lack of any significant decrease in stillbirth rates in developed country settings over the past 10 – 20 years [5].

As with other high income settings, the number of women delaying childbearing in Australia continues to increase. New South Wales (NSW) is the most populous state in Australia. The mean maternal age is 30.6 years; approximately 1 in 4 women (23%) deliver a baby aged 35 years or older and 15% of women have their first baby aged 35 or older [6]. Smoking during pregnancy in NSW has declined but remains at 12.7% in the overall population and particularly high at > 50% in women of Aboriginal or Torres Strait Islander origin [6]. Obesity is an increasing problem in pregnant women contributing not only to other pregnancy risks such as hypertension but also independently to stillbirth [7,8]. In 2009 40% of Australian women of child bearing age were either overweight or obese [9], and in 2008 60% of pregnant women in South Australia were documented as being overweight or obese [10]. Both smoking and obesity are risk factors modifiable by women who are planning a pregnancy however maternal age is not and the underlying mechanism of the age-associated risk remains unclear. A recent systematic review of maternal age and risk of stillbirth was unable to pool studies to determine the magnitude of stillbirth risk due to significant statistical heterogeneity [11]. Also, few of the included studies analysed antepartum stillbirths (before labour) separately to intrapartum stillbirths (during labour), a significant omission as the underlying causes, the associated risk factors and the suggested denominators for statistical analysis differ [12-14]. Although awareness of increased risks of adverse pregnancy outcome may contribute over time to women having children earlier there is a need to be able to give accurate risks to older women who are currently pregnant or planning a pregnancy. It is also important to be able to define risk of antepartum stillbirth by gestational age as for risks that are increased near term the option of timed/induced delivery is possible without the consequences of prematurity. Two very large recent studies in the US and Norway have assessed risk of stillbirth per ongoing pregnancy by gestational age week for different maternal age groups however neither was able to separate antepartum from intrapartum deaths or provide any cause of death information [15,16].

Despite known risk factors for stillbirth many of these deaths remain unexplained and there is an urgent need to identify and target appropriate areas for research and prevention of stillbirth. Unexplained deaths account for 41% of stillbirths in NSW and 28% nationally [17]. Stillbirths close to term are more likely to be classified as unexplained than very preterm stillbirths with 60% of term stillbirths in NSW classified as unexplained [17]. Not knowing why a baby died makes it difficult for health providers to counsel regarding future pregnancy management and recurrence risk and potentially magnifies the grief and bereavement outcomes for the family. Unexplained stillbirths have been shown in an analysis of stillbirths from Canada to be the only type of stillbirths statistically more common in older women [18] however the majority of studies assessing maternal age and stillbirth do not include information of cause of death [11].

One study addressing uteroplacental insufficiency and its association with advanced maternal age showed no difference in the stillbirths with fetal growth restriction between older and younger women [19].

The purpose of this study was to determine the most important current risk factors for antepartum stillbirth in NSW Australia and to quantify the risk of advanced maternal age by gestation in order to better inform older mothers and maternity care providers, particularly regarding risks at the end of pregnancy. Additionally we aimed to assess whether the classification of cause of death differed for stillbirths in older women.

## Methods

This was a State wide population based cohort study using de-identified linked data from two NSW data sets: the New South Wales Midwives Data Collection and the Perinatal Death Data from the NSW Maternal and Perinatal Committee. Ethical approval was obtained from the NSW Department of Health Ethics Committee Ref No DoHEC 2005-06-11.

### Data sources

The New South Wales Midwives Data Collection is a mandated population-based surveillance system covering all births in NSW public and private hospitals, as well as home births [6]. It encompasses all livebirths and stillbirths of at least 20 weeks gestation or at least 400 grams birth weight. The Midwives Data Collection requires the attending midwife or doctor to complete a notification form when a birth occurs, and collects demographic, maternal health, pregnancy, labour, delivery, and perinatal outcomes data (Additional file 1: Appendix S1). The NSW Maternal and Perinatal Committee is a quality assurance committee established under the NSW Health Administration Act 1982, and is privileged under this Act to carry out confidential reviews of both maternal and perinatal deaths [6]. Members are appointed by the Minister for Health. A subgroup called the Perinatal Outcomes Working Party reviews and classifies perinatal deaths using the Perinatal Society of Australia and New Zealand (PSANZ) – Perinatal Mortality Classification System which has been documented to have a high interobserver reliability with a kappa value of 0.83 – 0.95 [20,21]. Information available to the working party at review is forwarded by hospitals and includes a confidential report on perinatal death (Additional file 2: Appendix S2), post mortem and placental pathology reports as well as any other information considered relevant by the local hospital perinatal death review committee. Information considered by the Committee is confidential.

### Definitions

Gestational age was determined by certain dates confirmed by ultrasound before 20 weeks gestation or if dates uncertain by ultrasound alone prior to 20 weeks or if unavailable by examination of the newborn infant. Stillbirth was defined as a baby born of at least 20 weeks gestation or 400 g birthweight who did not, at any time after delivery, breathe, or show any evidence of life such as a heartbeat. Antepartum stillbirth was defined as death before the onset of labour from the Perinatal Death Database or induction of labour for known fetal death from the Midwives Data Collection. Unexplained antepartum death was defined as the death of a normally formed fetus prior to the onset of labour where no predisposing factors are considered likely to have caused the death. (PSANZ PDC classification of 10 or 11).Explained antepartum death was defined as a PSANZ – PDC classification of 2 – 9. Area Health Services and Country of birth grouping into English speaking and other regional groups are defined as per NSW Mothers and Babies Report [6].

### Study population

Records for babies born between 2002–2006 from the Midwives Data Collection and Perinatal Deaths data compiled by the NSW Maternal and Perinatal Committee subgroup were linked using probabilistic record linkage methods in the NSW Centre for Health Record Linkage. Babies were included if they were at least 22 weeks and less than 43 weeks gestation. Cause of Death classification was extracted from the Perinatal Death Database on all stillborn infants that had been reviewed by the Perinatal Outcomes Working Party. Perinatal Deaths classified as being due to congenital abnormality by the PSANZ Perinatal Death Classification system were excluded. This category includes deaths where a congenital abnormality (structural, functional or chromosomal) is considered to have made a major contribution to the death and includes terminations of pregnancy for congenital abnormality. Demographic data were collected from both datasets.

### Statistical analysis

Rates of antepartum stillbirth were computed by dividing the number of stillbirths by the sum of live births and stillbirths and multiplying by 1000. The risk of stillbirth was compared between groups by time to event analyses using gestation as the time scale and antepartum stillbirth as the event. All other births were censored. Crude and adjusted hazard ratios were estimated using a Cox proportional hazards model. We used Wald tests to test the effect of each covariate and those with P-values less than 0.05 were considered significant. Only first birth within the time period was included to avoid intraperson correlation. The proportional hazards assumption was tested using Schoenfeld residuals which were plotted against each covariate and the graphs inspected for any trend in the residuals [22,23]. Interactions between the log of gestational age and each covariate were created and added to the model individually to test for any departure from the proportional hazards assumption [24]. Records with missing data for the variables analysed were excluded. We calculated the risk of still birth at each week of gestation by dividing the number of still births that occurred during that week by the total number of ongoing pregnancies at the beginning of the week minus half of the number of live births in that week. All births were included in the calculation of these risks. Statistical analysis was performed using SAS version 9.2.

## Results

There were a total of 426086 singleton births of at least 22 weeks gestation in NSW over the five year study period excluding deaths due to fetal anomaly. Using only first birth within the time period there were 333099 births and 1161 antepartum stillbirths. 5409 records (1.6%) with missing data for the variables were excluded and final analysis was performed on 327690 births and 1127 antepartum stillbirths. Overall stillbirth rates are presented in Table 1.

**Table 1:** Antepartum stillbirth rate in New South Wales Australia 2002-2006

	**Births**	**Stillbirths**	**Rate per 1,000 births**
All births 2002-2006	426086	1438	3.4
- Only first birth for each woman in 2002-2006	333099	1161	3.5
- Excluding records with missing values for the variables analysed*	327690	1127	3.5
Analysed	327690	1127	3.5

* variables analysed = aboriginal, age, parity, pre-existing diabetes, pre-existing hypertension, smoking, sex of child, gestational diabetes, pre-eclampsia, area of residence.

### Risk factors for antepartum stillbirth

Crude and adjusted hazard ratios (HR) for antepartum stillbirth are presented in Table 2.

**Table 2:** Crude and adjusted risk factors for antepartum stillbirth in New South Wales Australia 2002 – 2006

		**Antepartum still births (ante=1)**	**Births**	**Univariable hazard ratio**	**95% confidence interval**	**Multivariable hazard ratio**	**95% confidence interval**
Indigenous							
	Aboriginal or Torres Strait Islander	35	8121	1.31	0.94, 1.84	1.09	0.77, 1.55
	Not ATSI	1092	319569				
Maternal age (years)							
	< 20	66	15221	1.31	0.98, 1.74	1.18	0.88, 1.57
	20 -24	161	48302	Referent	Referent		
	25-29	304	92003	1.00	0.83, 1.21	1.14	0.94, 1.34
	30-34	343	107730	0.98	0.81, 1.18	1.21	1.00, 1.47
	35-39	184	52748	1.09	0.88, 1.35	1.40	1.12, 1.75
	≥ 40	69	11686	1.90	1.43, 2.52	2.41	1.80, 3.23
Parity							
	Multiparous	502	154473	Referent			
	Nulliparous	625	173217	1.07	0.96, 1.21	1.23	1.08, 1.40
Preexisting diabetes							
	No	1110	325801	Referent			
	Yes	17	1889	3.11	1.93, 5.03	2.65	1.63, 4.32
Preexisting hypertension							
	No	1095	324194	Referent			
	Yes	32	3496	3.03	2.13, 4.31	2.77	1.94, 3.97
Smoking during pregnancy							
	No	877	279191	Referent			
	Yes	250	48499	1.68	1.46, 1.93	1.82	1.56, 2.12
Baby gender							
	Female	547	158448	Referent			
	Male	580	169242	1.00	0.89, 1.12	1.00	0.89, 1.12
Gestational diabetes							
	No	1088	312475	Referent			
	Yes	39	15215	0.80	0.58, 1.10	0.65	0.47, 0.91
Pre-eclampsia							
	No	1057	308508	Referent			
	Yes	70	19182	1.18	0.93, 1.50	1.13	0.88, 1.44
Area health service of residence							
	Sydney South West AHS	291	74122	1.26	1.05, 1.53	1.23	1.01, 1.49
	South Eastern Sydney & Illawarra AHS	169	54949	Referent			
	Sydney West AHS	239	62504	1.23	1.01, 1.50	1.17	0.96, 1.43
	Northern Sydney & Central Coast AHS	127	51340	0.80	0.64, 1.01	0.81	0.65, 1.02
	Hunter & New England AHS	139	37629	1.19	0.95, 1.49	1.16	0.92, 1.46
	North Coast AHS	58	18194	1.02	0.76, 1.38	0.99	0.73, 1.34
	Greater Southern AHS	43	14816	0.93	0.67, 1.30	0.90	0.65, 1.37
	Greater Western AHS	61	14136	1.40	1.04, 1.87	1.33	0.99,1.80
Mother’s Country of birth							
	English speaking	847	255602	Referent			
	Central and South America	8	2862	0.85	0.42, 1.71	0.90	0.45, 1.81
	Eastern Europe, Russia, Central Asian and Baltic States	12	2136	1.68	0.95, 2.97	1.84	1.04, 3.25
	Melanesia, Micronesia and Polynesia	32	5801	1.67	1.17, 2.38	1.70	1.19, 2.44
	Middle East and Africa	82	14768	1.64	1.31, 2.06	1.74	1.38, 2.21
	North East Asia	27	12637	0.65	1.44, 0.96	0.72	0.49, 1.06
	South East Asia	58	18592	0.98	0.75, 1.28	1.05	0.80, 1.38
	Southern Asia	44	8802	1.53	1.13, 2.07	1.75	1.28, 2.39
	Southern Europe	14	3836	1.10	0.65, 1.87	1.10	0.65, 1.87
	Western and Northern Europe	3	2654	0.34	0.11, 1.04	0.35	0.11, 1.10

Risk factors significantly associated with antepartum stillbirth on adjusted analyses were: maternal age, smoking, nulliparity, pre-existing hypertension, pre-existing diabetes, area health service of residence and country of birth. Maternal age showed a dose–response relationship with a HR 1.4 (95% confidence interval (CI) 1.12, 1.75) for women aged 35 – 39 and HR 2.41 (95% CI 1.8, 3.23) for women aged 40 and above. Smoking conferred an increased risk of 1.82 (95% CI 1.56, 2.12), nulliparity 1.23 (95% CI 1.08, 1.40), preexisting diabetes 2.65 (95% CI 1.63, 4.32) and pre existing hypertension 2.77 (95% CI 1.94, 3.97). Area health service of residence and country of birth were used as available surrogate markers of socioeconomic status. There was an increased risk of antepartum stillbirth for women residing in Sydney South West Area Health Service (HR 1.23 95% CI 1.01,1.49) This represents an area with both a large multicultural population and the largest number of births in NSW. There were also increased risks of antepartum stillbirth for women born in Eastern Europe, Russia and the Baltic States (HR 1.84 95% CI 1.04, 3.25) Melanesia, Micronesia and Polynesia (HR1.7 95% CI 1.19, 2.44) Middle East and Africa (HR1.74 95% CI 1.38, 2.21) and Southern Asia (HR1.75 95% CI 1.28, 2.39). Importantly, after adjusting for the other variables indigenous status was not a significant risk factor for antepartum stillbirth (HR 1.09 95% CI 0.77, 1.55). Gestational diabetes on adjusted analysis appeared to reduce the risk of antepartum stillbirth (HR 0.65 95% CI 0.47, 0.91).

### Risk factors stratified by parity

Adjusted risk factors stratified by parity are shown in Table 3. Multiparous women whose previous birth was by caesarean section had an increased risk of antepartum stillbirth that was close to but did not reach statistical significance (HR 1.24 95% CI 0.99,1.55). The dose response relationship with maternal age for increased antepartum stillbirth risk was seen for both nulliparous and multiparous women but with slightly larger effect sizes in the nulliparous women. The increased risk seen overall for women residing in Sydney South Western Area Health Service is only significant for multiparous women which may reflect referral patterns into tertiary centres for known higher risk pregnancies. For women from Middle East and Africa there remains increased risk despite parity. The increased risk for women from Melanesia, Polynesia and Micronesia and Russia and the Baltic states was only seen for multiparous women and conversely only in nulliparous women from Southern Asia, and may be a reflection of the small numbers.

**Table 3:** Adjusted risk factors for antepartum stillbirth by parity in New South Wales Australia 2002 – 2006

	**Nulliparous women Ante SB Births**		**AHR**	**95% confidence interval**	**Multiparous women Ante SB Births**		**Adj HR**	**95% confidence interval**
Indigenous								
Aboriginal or Torres Strait Islander	15	3586	0.97	0.57, 1.67	20	4535	1.19	0.74, 1.90
Not ATSI	610	169631	Ref		482	149938	Ref	
Maternal age (years)								
< 20	63	14002	1.23	0.90, 1.68	3	1219	0.77	0.24, 2.47
20 -24	117	33401	Ref		44	14901	Ref	
25-29	178	53542	1.07	0.84, 1.35	126	38461	1.22	0.86, 1.72
30-34	167	50180	1.16	0.90, 1.48	176	57550	1.26	0.90, 1.76
35-39	75	18510	1.45	1.08, 1.96	109	34238	1.36	0.95, 1.94
≥ 40	25	3582	2.58	1.67, 4.01	44	8104	2.32	1.52, 3.56
Preexisting diabetes								
No	616	172342	Ref		494	153459	Ref	
Yes	9	875	3.06	1.57, 5.97	8	1014	2.20	1.08, 4.50
Preexisting hypertension								
No	611	171422	Ref		484	152772	Ref	
Yes	14	1795	2.25	1.31, 3.85	18	1701	3.38	2.09, 5.49
Smoking during pregnancy								
No	511	152057	Ref		366	127134	Ref	
Yes	114	21160	1.71	1.37, 2.13	136	27339	1.93	1.56, 2.38
Baby gender								
Female	287	83575	Ref		260	74873	Ref	
Male	338	89642	1.11	0.95, 1.30	242	79600	0.88	0.74, 1.05
Gestational diabetes								
No	607	166321	Ref		481	146154	Ref	
Yes	18	6896	0.66	0.41, 1.06	21	8319	0.65	0.42, 1.02
Pre-eclampsia								
No	578	160272	Ref		479	148236	Ref	
Yes	47	12945	1.10	0.82, 1.49	23	6237	1.15	0.76, 1.76
Area health service of residence								
Sydney South West AHS	151	39377	1.13	0.88, 1.45	140	34745	1.39	1.02, 1.88
South Eastern Sydney & Illawarra AHS	106	31893	Ref		63	23056	Ref	
Sydney West AHS	135	32100	1.19	0.92, 1.54	104	30404	1.16	0.84, 1.59
Northern Sydney & Central Coast AHS	78	28913	0.80	0.60, 1.07	49	22427	0.83	0.57, 1.20
Hunter & New England AHS	77	18615	1.19	0.88, 1.61	62	19014	1.14	0.80, 1.63
North Coast AHS	30	8621	0.98	0.65, 1.48	28	9573	1.00	0.64, 1.57
Greater Southern AHS	21	7024	0.85	0.53, 1.37	22	7792	0.97	0.60, 1.59
Greater Western AHS	27	6674	1.14	0.74, 1.75	34	7462	1.56	1.02, 2.39
Mother’s Country of birth								
English speaking	483	136476	Ref		364	119126	Ref	
Central and South America	4	1556	0.77	0.29, 2.07	4	1306	1.07	0.40, 2.89
Eastern Europe, Russia, Central Asian and Baltic States	5	1343	1.15	0.48, 2.78	7	793	3.27	1.54, 6.94
Melanesia, Micronesia and Polynesia	11	2320	1.42	0.78, 2.59	21	3481	1.92	1.22, 3.01
Middle East and Africa	33	6049	1.60	1.11, 2.29	49	8719	1.86	1.35, 2.55
North East Asia	19	7338	0.81	0.51, 1.29	8	5299	0.57	0.28, 1.15
South East Asia	32	9898	1.03	0.71, 1.49	26	8694	1.08	0.71, 1.63
Southern Asia	30	4843	2.00	1.37, 2.93	14	3959	1.36	0.79, 2.35
Southern, Western and Northern Europe	8	3394	0.68	0.34, 1.37	9	3096	0.96	0.49, 1.87
Previous birth by CS								
No					402	125257	Ref	
Yes					100	29216	1.24	0.99, 1.55

### Risk of antepartum stillbirth with advanced maternal age

**Figure 1:**
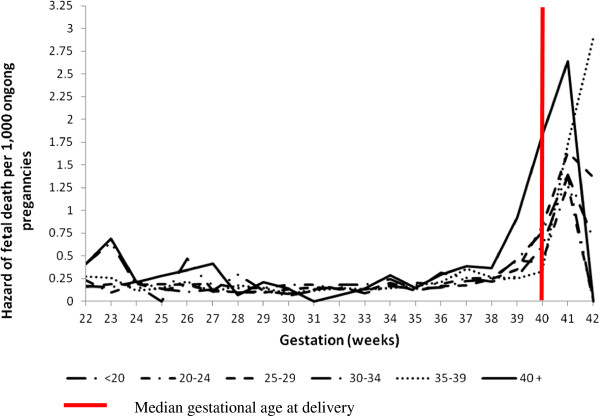
**Risk of antepartum stillbirth by gestational age and maternal age group New South Wales Australia 2002 – 2006.** Median gestational age at delivery.

Gestational age specific risk of antepartum stillbirth is shown in Figure 1. The risk of antepartum stillbirth per undelivered pregnancy rises from around 40 weeks for all women. Importantly this increased risk is apparent at earlier gestation by 1 – 2 weeks for women aged 40 and above. Absolute risks per 1000 undelivered pregnancies by parity for all age groups beyond 40 weeks of pregnancy are presented in Table 4. The overall absolute risk of stillbirth beyond 40 weeks gestation for women aged 40 and above was 2.2 per 1000 or 1 in 455 ongoing pregnancies compared with 0.85 per 1000 or 1 in 1177 ongoing pregnancies for women under 40. The risk is highest for nulliparous women aged 40 and above with 1 in 247 ongoing pregnancies resulting in an antepartum stillbirth.

**Table 4:** Absolute risks of stillbirth ≥ 40 weeks gestation per 1000 undelivered pregnancies by maternal age and parity, New South Wales Australia 2002 - 2006

**Age group**	**Antepartum stillbirths**	**Undelivered pregnancies**	**Risk per 1000 undelivered pregnancies**
Nulliparous women			
< 20	6	7624	0.79
20 – 24	7	9174	0.76
25 – 29	32	28685	1.11
30 – 34	23	26366	0.87
35 – 39	13	9144	1.42
≥ 40	6	1481	4.05
Multiparous women			
< 20	1	1207	0.83
20 – 24	13	12204	1.07
25 – 29	27	27397	0.99
30 – 34	34	35603	0.95
35 – 39	7	18480	0.38
≥ 40	5	3513	1.42

### Unexplained versus explained deaths

PSANZ Classification of Cause of Death stratified by both parity and maternal age of 40 or above is shown in Table 5. Standardised Classifications from the Perinatal Outcomes Working Party were available for 1016 of the 1127 antepartum stillbirths (90%). The most common classification of death was unexplained (549/1016). Adjusted analyses for risk factors were performed for the unexplained group and showed similar hazard ratios for all variables to the total antepartum group so are not presented here. Adjusted analyses were not performed for the explained causes of death as the numbers in each group became too small. Multiparous women were more likely to have a classification of death of antepartum haemorrhage, maternal or perinatal conditions and nulliparous women more likely to have their baby’s death classified as due to growth restriction. The proportions of unexplained deaths were high in both groups. There was no difference in the proportion of deaths classified as fetal growth restriction between women aged 40 or above compared with the younger women.Women aged 40 or above were more likely to have their baby’s death classified as due to perinatal infection or spontaneous preterm, although the numbers within these groups are small.

**Table 5:** Classification of cause of death by parity and maternal age in New South Wales Australia 2002 – 2006

**PSANZ Classification**	**No. (%)**	**Parity Nulliparous Multiparous**		**Maternal age 40 and above < 40 years**	
Unexplained Antepartum Death	546 (54)	304 (53.6)	242 (53.9)	516 (54)	30 (49)
Spontaneous Preterm	84 (8.3)	48 (8.5)	36 (8.0)	74 (7.8)	10 (16.4)
Antepartum Haemorrhage	76 (7.5)	30 (5.3)	46 (10.2)	76 (7.9)	0
Maternal Conditions	66 (6.5)	31 (5.4)	35 (7.8)	63 (6.6)	3 (4.9)
Hypertension	65 (6.4)	49 (8.6)	16 (3.6)	59 (6.2)	6 (9.8)
Fetal Growth Restriction	62 (6.1)	43 (7.6)	19 (4.2)	58 (6.1)	4 (6.6)
Specific Perinatal Conditions	58 (5.7)	26 (4.6)	32 (7.1)	55 (5.8)	3 (4.9)
Perinatal Infection	38 (3.7)	22 (3.9)	16 (3.6)	34 (3.6)	4 (6.5)
Hypoxic Peripartum Death	18 (1.8)	12 (2.1)	6 (1.3)	17 (1.8)	1 (1.6)
No Obstetric Antecedent	3 ( 0.3)	2 (0.3)	1 (0.2)	3 (0.3)	0
Totals.	1016	449	567	955	61

## Discussion

This represents a large cohort of antepartum stillbirths using record linked data and is the first study to specifically examine risk factors for antepartum stillbirth by undelivered pregnancy in an Australian population. We have confirmed that increasing maternal age and nulliparity are important demographic risk factors for antepartum stillbirth in New South Wales. We have also been able to document the risk per undelivered pregnancy by gestational age week for maternal age strata and present absolute risks at term and beyond for older women. These findings will be important to both pregnant women and their clinicians in the provision of accurate information regarding stillbirth risk and timing.

In this setting smoking, pre-existing diabetes and pre-existing hypertension also remain important independent (and potentially modifiable) risk factors for antepartum stillbirth. Smoking rates are extremely high in the Indigenous population in Australia [6] and represent a particular opportunity for stillbirth prevention where evidence of benefit for cessation strategies exists [25]. The increased risk often attributed solely to Indigenous status is not significant after adjusting for the other variables collected and is evidence that health care provision and access as well as health promotion strategies in pregnancy could be better targeted to these women. Further evidence of disparity appears to exist with our finding that women from non-English speaking backgrounds particularly the Middle East and Africa have higher independent risks of stillbirth in the Australian setting. Whilst we are unable to examine the underlying reasons for this further with this data this is an issue that is widely generalisable to similar high income countries with immigrant minority communities. In such settings it will remain important to ensure equal access and opportunities for the provision of maternity care to all women regardless of background.

The finding that gestational diabetes was apparently protective for antepartum stillbirth on adjusted analyses may relate to a number of factors. It is possible that identified women with this risk factor are receiving more regular antenatal care and monitoring and have therefore a reduced risk. They are also more likely to be induced and less likely to go post term. In this cohort 9.8 % of women with gestational diabetes delivered at 41 weeks gestation or beyond compared with 20.3% of the total sample. It may also be possible that we have been unable to adjust for other confounders not collected in population data. Recent population based studies have actually shown no increased risk for stillbirth for women with gestational diabetes [26,27] with further evidence of low fetal death rates seen in the prospective Hyperglycaemia and Adverse Pregnancy Outcome (HAPO) cohort [28] (stillbirth rate of 3.8 per 1000) and no stillbirths in the intervention arm in the Australian Carbohydrate Intolerance Study in Pregnant Women (ACHOIS) study compared with standard care arm (stillbirth rate of 5.7 per 1000) [29].

Strengths of our data include the large population base, the very small proportion of missing data (1.6%) and the standardised classification of cause of death. The Midwives Data Collection has previously performed very well in validation studies [30]. We have ascertained timing of death from two independent data sources and are confident of the accuracy of the antepartum coding. This meant we did not have to rely on reporting of Apgar scores which are subjective, have high interobserver variability and are often subject to miscoding [31,32]. It is possible however that our focus on accuracy of timing may have reduced the overall antepartum stillbirth numbers assessed as our rates of 3.4 per 1000 are slightly lower than other reported rates from similar populations of approximately 4.0 per 1000 [5].

The quantification of gestational-specific risk of antepartum stillbirth by maternal age has not previously been documented in an Australian setting and represents significant new information for maternity health care providers. Although absolute risks for older women are small, they are consistent with risks of chromosomal abnormalities in the magnitude of 1/192 – 1/378 [33] within the same group of women for which they currently receive considerable counselling and potentially subsequent investigations. Women aged 35 and above and particularly those aged 40 or more should receive some information regarding stillbirth risk and be able to discuss potential options with their health care provider to assist with informed decision making. Ideally the question of balancing benefits and harms of induction of labour at term versus post term needs to be answered in a large randomised controlled trial. This may not be feasible as numbers needed would exceed 46,000 women in order to show a 1 per 1000 difference in late pregnancy stillbirth. Further, the balance of harms in the intervention group is complicated as the comparison of a short nursery admission with respiratory problems compared with a baby dying in utero are neither going to be comparable, nor acceptable to women. One randomised study has used statistical modelling of individual risk factors in women aged 35 or greater to estimate an optimal timing of delivery at term and induce women in the intervention group who had not entered spontaneous labour by this time [34]. Although it was not powered to assess mortality there were reduced adverse outcomes including nursery admission and caesarean section in the intervention arm.

Having access to standardised classification of cause of death for 90% of the stillborn babies analysed is rare for large population based studies and this linkage has allowed us to both exclude deaths due to congenital anomalies and examine cause of death relative to the maternal perinatal risk factors of parity and maternal age. The classification of cause of death uses the PSANZ Perinatal Death Classification system which has been endorsed nationally and has now also been shown to perform well against other classification systems used internationally [35]. Although small numbers did not permit adjusted analyses for the explained causes of death for women aged 40 and above it is interesting to note that deaths due to perinatal infection were more common in these women. This is consistent with recent literature documenting increased incidence of placental histological evidence of infection in term stillbirths [36,37] and suggests a potential area for future research into underlying mechanisms.

There are however inherent shortcomings in using routinely collected population based datasets. The detail that can be obtained is limited to what information is collected and ascertainment of stillbirth is less accurate for extremely premature gestations [38]. Importantly, maternal height and weight, or BMI are not collected in the perinatal population health databases in New South Wales so we are unable to assess the importance of this risk factor on antepartum stillbirth. As a significant increasing public health problem and independent contributor to poor pregnancy outcomes there is an argument to be made to add this information to population perinatal data collection throughout Australia. We also do not have information on the timing of death just the timing of delivery which may affect the proportion of deaths classified as due to fetal growth restriction.

## Conclusion

In summary we have confirmed the most important risk factors on a population basis for antepartum stillbirth in NSW and have quantified the risk of advanced maternal age by gestation. Women aged ≥ 35 in a first pregnancy should receive information regarding stillbirth risk. For those women at highest risk, that is age > 40 and in their first pregnancy it would be reasonable to offer induction of labour if undelivered by 40 weeks rather than waiting until 41 weeks or beyond. Large international randomised controlled trials are required to answer this question however these may be neither feasible or timely. Future studies therefore need to focus on the benefits and harms of any practice change in this area.

## Abbreviations

NSW: New South Wales; PSANZ: Perinatal Society of Australia and New Zealand. A multidisciplinary society that includes: scientists, obstetricians, midwives, neonatologists, neonatal nurses, epidemiologists, surgeons, therapists and other professionals focussed on perinatal medicine. The Society covers basic science through to clinical practice and offers professional, political, administrative and educational advice in the area of perinatal medicine; AHS: area health service; AHR: adjusted hazard ratio.

## Competing interests

The authors have no conflict of interest to declare.

## Authors' contributions

AG designed the study, linked the datasets, extracted data and drafted the final paper. CRG contributed to data linkage, data analysis and interpretation and drafting the paper. KMG performed statistical analysis and commented on the paper. HJ provided overall supervision and advice. JM provided general advice and comments on the paper. All authors reviewed the study findings and read and approved the final manuscript.

## Pre-publication history

The pre-publication history for this paper can be accessed here:

http://www.biomedcentral.com/1471-2393/13/12/prepub

## Supplementary Material

Additional file 1 **Appendix S1.** Midwives Data Collection. Click here for file

Additional file 2 **Appendix S2.** Confidential Report on Perinatal Death. Click here for file
