# Supplementary material for: Risk factors for antepartum stillbirth and the influence of maternal age in New South Wales Australia: A population based study
Source: BMC Pregnancy Childbirth. 2013 Jan 16;13:12. doi: 10.1186/1471-2393-13-12 (PMC3552834; doi:10.1186/1471-2393-13-12)
Supplement: Additional file 1 — Appendix S1. Midwives Data Collection. [file 1471-2393-13-12-S1.pdf]

Appendix S1: Midwives Data Collection

NSW MIDWIVES DATA COLLECTION

Mother Unit Record No. **Hospital** **Code**

First Name **Family Name**

Address **Postcode**

Mother's birth date day month year

Country of birth Australia 36 Other

If other, specify

Indigenous status: Aboriginal 1 Torres Strait Islander 2 Aboriginal and Torres Strait Islander 3 None of the above 4

PREVIOUS PREGNANCIES

Previous pregnancy greater than 20 weeks? Yes 1 No 0

If no, go to next section.

If yes:

Specify the number of previous pregnancies > 20 weeks

Was the last birth by caesarean Yes 1 No 0

Total number of previous caesarean sections?

THIS PREGNANCY

Date of LMP day month year

Prenatal diagnosis (< 20 weeks gestation) CVS Amniocentesis

Antenatal care

Duration of pregnancy at first visit (weeks)

Not booked

Medical conditions

Diabetes mellitus

Gestational diabetes

Chronic hypertension

Pre-eclampsia

Smoking

Did the mother smoke at all during pregnancy? Yes 1 No 0

If yes, how many cigarettes each day on average in the second half of pregnancy?

None 1 > 10 per day 2 ≤ 10 per day 3 Unknown 4

LABOUR AND DELIVERY

Onset of labour

Spontaneous 1 Induced 2 No labour 3

If labour augmented/ induced (tick 1 or more):

Oxytocins ARM Prostaglandins Other

LABOUR AND DELIVERY

If labour induced, main indication:

Diabetes 1

Hypertensive disease 2

Fetal distress 3

Fetal death 4

Chorioamnionitis 5

Blood group isoimmunisation 6

Prelabour rupture of membranes 7

Prolonged pregnancy (41+ weeks) 8

Suspected intrauterine growth restriction 9

Other 10

Pain relief/ anaesthetics *(tick 1 or more)*

None Pudendal Nitrous oxide Spinal IM narcotics General anaesthetic Local to perineum Epidural/caudal Other

Presentation at birth

Vertex 1 Face 3 Breech 2 Brow 4 Other 5

Type of delivery

Normal vaginal 1 Vacuum extr. 3 Forceps 2 Vaginal breech 4 Caesarean section 5

If caesarean section, main indication:

Failure to progress

- Cx dilatation unknown 1
- Cx 3cm dilated or less 2
- Cx dilated more than 3 cm 3

Fetal distress 4

Other 5

Perineal status

Intact 1 4th deg. tear 5 1st deg. tear/graze 2 Episiotomy 6 2nd deg. tear 3 Both tear and episiotomy 7 3rd deg. tear 4 Other 8

Surgical repair of the vagina or perineum? Yes 1 No 0

BABY

Place of birth

Hospital theatre/delivery suite 1

Birth centre 2

Planned birth centre/delivery suite birth 3

Planned homebirth 4

Planned homebirth/hospital admission 5

Born before arrival 6

Unit Record No.

Birth date: day month year

Sex: M 1 F 2 Indet. 3

Plurality: Single 1 Multiple 2

If multiple, total number

If multiple birth, specify baby number

Birthweight (grams)

Estimated gestational age

Apgar 1 min 5 min

Resuscitation of baby *(tick 1 or more)*

None 1 IPPR : bag + mask 4 Suction 2 Intubation + IPPR 5 O2 therapy 3 External cardiac massage + ventilation 6 Other 7

POSTNATAL CARE - BABY

Birth defect? Yes 1 No 0

If yes, specify:

Admitted to NICU? Yes 1 No 0

Admitted to SCN? Yes 1 No 0

If yes, observation only? Yes 1 No 0

If admitted to SCN/NICU:

Was a birth defect the main reason for admission? Yes 1 No 0

DISCHARGE STATUS - MOTHER AND BABY

Mother

Discharged 1 Transferred 2 Died 3

Baby

Discharged 1 Transferred 2 Stillbirth 3 Neonatal death 4 Transferred and died 5

Baby's date of discharge or transfer day month year

Hospital transferred to:

If baby died, date of death day month year

Signature of midwife at discharge

Health Department Copy

Please complete and forward to: NSW Midwives Data Collection
Patient Data Management Unit, Level 6
Locked Bag 961, North Sydney, NSW 2059
