# Supplementary material for: Risk factors for antepartum stillbirth and the influence of maternal age in New South Wales Australia: A population based study
Source: BMC Pregnancy Childbirth. 2013 Jan 16;13:12. doi: 10.1186/1471-2393-13-12 (PMC3552834; doi:10.1186/1471-2393-13-12)
Supplement: Additional file 2 — Appendix S2. Confidential Report on Perinatal Death. [file 1471-2393-13-12-S2.pdf]

Appendix S2: Confidential Report on Perinatal Death

NSW MATERNAL AND PERINATAL COMMITTEE
CONFIDENTIAL REPORT ON PERINATAL DEATH

Office use: Reference No.:

HOSPITAL: ____________________

MOTHER DETAILS:

Mother's name ____________________

Address: ____________________

____

Medical Record No.: ☐☐☐☐☐☐☐☐☐☐

BABY DETAILS:

Baby's name: ____________________

Type of perinatal death: Stillbirth ☐ Neonatal death ☐

Date of birth/ stillbirth: / /

If liveborn: Date of death: / /

Age at death: day(s) hour(s)

Medical Record No.: ☐☐☐☐☐☐☐☐☐☐

For STILLBIRTHS complete Part A and for NEONATAL DEATHS complete Parts A and B

PART A

1. Was a postmortem examination carried out?

Yes ☐ No ☐ Unknown ☐

If yes, please include a copy of the report.

2. Was histopathological examination of the placenta carried out?

Yes ☐ No ☐ Unknown ☐

If yes, please include a copy of the report.

If no: Placental weight: ☐☐☐ grams

Describe the placental appearance:

____

____

3. Is this baby one of a multiple pregnancy (twin, triplet etc)?

Yes ☐ No ☐ Unknown ☐

If yes: Number of babies ☐☐

Birth order ☐☐

4. Bleeding during pregnancy?

Yes ☐ No ☐ Unknown ☐

If yes: Threatened miscarriage ☐

Placental abruption ☐

Placenta praevia ☐

Vasa praevia ☐

Undetermined ☐

Other ☐

If other, specify:

____

5. Was hypertension present?

Yes ☐ No ☐ Unknown ☐

If yes: Chronic hypertension:

Essential ☐

Secondary eg renal disease ☐

Unspecified ☐

Gestational hypertension ☐

Pre-eclampsia ☐

Chronic + superimposed pre-eclampsia ☐

Unspecified ☐

6. Any other maternal diseases present in pregnancy?

Yes ☐ No ☐ Unknown ☐

If yes: Maternal injury:

Accidental ☐

Non-accidental ☐

Diabetes/gestational diabetes ☐

Sepsis ☐

Other ☐

If other, specify:

____

7. Was the death an unexplained antepartum death?

Yes ☐ No ☐ Unknown ☐

8. When did the death occur?

Before the onset of labour ☐

During labour ☐

Before birth, unknown time ☐

After birth ☐

9. Was there fetal growth restriction (weight less than 10th percentile)?

Yes ☐ No ☐ Unknown ☐

Note: for stillbirths who died before the onset of labour, serial U/S evidence of FGR is required.

10. Was there spontaneous preterm delivery (less than 37 weeks)?

Yes ☐ No ☐ Unknown ☐

If yes, what was the duration of rupture of membranes prior to delivery?

Less than 24 hours ☐

24 hours or more ☐

Unknown ☐

11. Was there intrapartum asphyxia?

Yes ☐ No ☐ Unknown ☐

12. Gestational age: ☐☐ weeks

13. Birthweight: ☐☐☐☐ grams

14. Sex: Male ☐

Female ☐

Indeterminate ☐

15. Onset of labour:

Spontaneous ☐

Induced ☐

No labour ☐

16. Type of delivery:

Normal vaginal delivery ☐

Breech delivery ☐

Caesarean section ☐

Forceps delivery ☐

Ventouse delivery ☐

Other delivery ☐

If other delivery, specify:

____

PART A (continued)

17. Were there cord complications?
Yes ☐ No ☐ Unknown ☐
If yes, describe:

18. Was a major fetal abnormality present?
Yes ☐ No ☐ Unknown ☐
If yes, describe:

19. Was chorioamnionitis present?
Yes ☐ No ☐ Unknown ☐
If yes, diagnosis was:
- Pathological ☐
- Clinical ☐

If yes, specify organism:

20. Infant/ fetal infection?
Yes ☐ No ☐ Unknown ☐
If yes:
- Streptococcus Group B ☐
- E Coli ☐
- Listeria monocytogenes ☐
- Cytomegalovirus ☐
- Parvovirus ☐
- Herpes simplex virus ☐
- Rubella virus ☐
- Toxoplasma ☐
- Syphilis ☐
- Other ☐

If other, specify:

21. Other conditions?
Yes ☐ No ☐ Unknown ☐
If yes:
- Twin-to-twin transfusion ☐
- Fetomaternal haemorrhage ☐
- Uterine abnormality ☐
- Birth trauma ☐
- Haemolytic disease ☐
- Idiopathic hydrops ☐
- Drug dependence/abuse ☐
- Termination of pregnancy ☐
- Other ☐

If other, specify:

22. Classification of obstetric cause of death (see Attachment)

☐ .

PART B (Neonatal deaths only)

THE MAIN CAUSE OF DEATH (tick one):

1. Congenital abnormality ☐

2. Extreme prematurity ☐
If yes, was resuscitation carried out?
Yes ☐ No ☐ Unknown ☐

3. Cardio-respiratory
- Hyaline membrane disease ☐
- Meconium aspiration syndrome ☐
- Primary persistent pulmonary hypertension ☐
- Pulmonary hypoplasia ☐
- Chronic neonatal lung disease ☐
- Other ☐

If other, specify:

4. Infection
- Congenital bacterial ☐
- Acquired bacterial ☐
- Congenital viral ☐
- Acquired viral ☐
- Protozoal eg Toxoplasma ☐
- Spirochaetal eg Syphilis ☐
- Fungal ☐
- Other ☐

If other, specify:

5. Neurological
- Hypoxic ischaemic encephalopathy/ perinatal asphyxia ☐
- Intracranial haemorrhage ☐
- Other ☐

If other, specify:

6. Gastrointestinal
- Necrotising enterocolitis ☐
- Other ☐

If other, specify:

7. Other
SIDS:
- Consistent with SIDS ☐
- Possible SIDS ☐

Multisystem failure (only if unknown primary cause or trigger event ☐
Trauma ☐
Undetermined ☐
Other ☐
If other, specify:

MAIN CAUSE OF DEATH AND RELEVANT FACTORS

Main cause of death as determined by hospital perinatal death review committee

Relevant factors:

Antenatal:

Intrapartum:

Postpartum:

Form completed by:
Name:

Date: / /

DEFINITION: FETAL GROWTH RESTRICTION

Less than the 10th percentile for gestation.

Gestation (weeks)	Weight (grams) 10th percentile Male	Female
22	400	400
23	500	470
24	520	540
25	620	620
26	720	680
27	740	730
28	850	760
29	950	890
30	1080	1045
31	1310	1140
32	1400	1340
33	1640	1520
34	1840	1760
35	2110	2030
36	2320	2220
37	2550	2430
38	2780	2660
39	2940	2820
40	3070	2950
41	3180	3050
42	3210	3080
43	3080	2950
44	3050	2930

Source: Roberts CL, Lancaster PAL. Australian national birthweight percentiles by gestational age. *Med J Aust* 1999; 170: 114-118.

Australia and New Zealand Antecendent Classification of Perinatal Mortality

1. Congenital abnormality
(including termination of pregnancy for congenital abnormality)
- 1.1 Central nervous system
- 1.2 Cardiovascular system
- 1.3 Urinary tract
- 1.4 Gastrointestinal tract
- 1.5 Chromosmal
- 1.6 Metabolic
- 1.7 Multiple
- 1.8 Other
- 1.9 Unspecified

2. Perinatal infection
- 2.1 Bacterial
  - 2.11 Group B Streptococcus
  - 2.12 E Coli
  - 2.13 Listeria Monocytogenes
  - 2.18 Other bacterial
  - 2.19 Unspecified bacterial
- 2.2 Viral
  - 2.21 Cytomegalovirus
  - 2.22 Parvovirus
  - 2.23 Herpes simplex virus
  - 2.24 Rubella virus
  - 2.28 Other viral
  - 2.29 Unspecified viral
- 2.3 Protozoal eg Toxoplasma
- 2.4 Spirochaetal eg Syphilis
- 2.5 Fungal
- 2.6 Other
- 2.7 Unspecified organism

3. Hypertension
- 3.1 Chronic hypertension: essential
- 3.2 Chronic hypertension: secondary eg renal disease
- 3.3 Chronic hypertension: unspecified
- 3.4 Gestational hypertension
- 3.5 Pre-eclampsia
- 3.6 Pre-eclampsia superimposed on pre-existing hypertension
- 3.7 Unspecified hypertension

4. Antepartum haemorrhage
- 4.1 Placental abruption
- 4.2 Placenta praevia
- 4.3 Vasa praevia
- 4.8 Other APH
- 4.9 APH of undertermined origin

5. Maternal disease
- 5.1 Termination of pregnancy (other than for fetal abnormality)
- 5.2 Diabetes / gestational diabetes
- 5.3 Maternal injury
  - 5.31 Accidental
  - 5.32 Non-accidental
- 5.4 Maternal sepsis
- 5.5 Other maternal conditions eg Lupus obstetric syndrome

6. Specific perinatal conditions
- 6.1 Twin-to-twin transfusion
- 6.2 Fetomaternal haemorrhage
- 6.3 Antepartum cord complications
- 6.4 Uterine abnormality
- 6.5 Birth trauma (typically >24 weeks or > 600 grams)
- 6.6 Haemolytic disease
- 6.7 Idiopathic hydrops
- 6.8 Other

7. Hypoxic peripartum death (typically > 24 weeks or > 600 grams)
- 7.1 With intrapartum complications
  - 7.11 Uterine rupture
  - 7.12 Cord prolapse
  - 7.13 Shoulder dystocia
  - 7.18 Other
- 7.2 No intrapartum complications
- 7.9 Unspecified hypoxic peripartum death

8. Fetal growth restriction (FGR)
- 8.1 With evidence of uteroplacental insufficiency eg significant infarction, acute atherosis, maternal vascular thrombosis or maternal floor infarction
- 8.2 With chronic villitis
- 8.3 Without the above placental pathology
- 8.4 No examination of placenta
- 8.9 Unspecified FGR or not known whether placenta examined

9. Spontaneous preterm
- 9.1 Spontaneous preterm with intact membranes, or membrane rupture less than 24 hours before delivery,
  - 9.11 with chorioamnionitis
  - 9.12 without chorioamnionitis
  - 9.13 no examination of the placenta
  - 9.19 unspecified or not known whether placenta examined
- 9.2 Spontaneous preterm with membrane rupture >= 24 hours before delivery,
  - 9.21 with chorioamnionitis
  - 9.22 without chorioamnionitis
  - 9.23 no examination of the placenta
  - 9.29 unspecified or not known whether placenta examined
- 9.3 Spontaneous preterm with membrane rupture of unknown duration before delivery,
  - 9.31 with chorioamnionitis
  - 9.32 without chorioamnionitis
  - 9.33 no examination of the placenta
  - 9.39 unspecified or not known whether placenta examined

10. Unexplained antepartum death
- 10.1 With evidence of uteroplacental insufficiency eg significant infarction, acture atherosis, maternal vascular thrombosis or maternal floor infarction.
- 10.2 With chronic villitis
- 10.3 Without the above placental pathology
- 10.4 No examination of placenta
- 10.9 Unspecified unexplained antepartum death or not known whether placenta examined.

11. No obstetric antecendent
- 11.1 SIDS
  - 11.11 Consistent with SIDS
  - 11.12 Possible SIDS
- 11.2 Postnatally acquired infection
- 11.3 Accidental asphyxiation
- 11.4 Other accident, poisoning or violence (postnatal)
- 11.8 Other
- 11.9 Unknown / Unexplained
